# Frailty and subsequent adverse outcomes in older patients with atrial fibrillation treated with oral anticoagulants: The Shizuoka study

**DOI:** 10.1016/j.rpth.2023.100129

**Published:** 2023-03-25

**Authors:** Shiori Nishimura, Hiraku Kumamaru, Satoshi Shoji, Eiji Nakatani, Hiroyuki Yamamoto, Nao Ichihara, Alexander T. Sandhu, Yoshiki Miyachi, Hiroaki Miyata, Shun Kohsaka

**Affiliations:** 1 Department of Healthcare Quality Assessment, The University of Tokyo Graduate School of Medicine, Tokyo, Japan; 2 Shizuoka Graduate University of Public Health, Shizuoka, Japan; 3 Department of Cardiology, Keio University School of Medicine, Tokyo, Japan; 4 Department of Health Policy and Management, Keio University School of Medicine, Shinjuku, Tokyo, Japan; 5 Division of Cardiovascular Medicine, Stanford University School of Medicine, Stanford, California, USA

**Keywords:** anticoagulants, atrial fibrillation, frailty, hemorrhage, stroke

## Abstract

**Background:**

In older patients with atrial fibrillation (AF), frailty is frequently prevalent. However, the prognostic value of frailty for adverse events after initiation of oral anticoagulants (OACs) is unclear.

**Objectives:**

We assessed whether frailty at the time of OAC initiation is associated with subsequent bleeding or embolic events.

**Methods:**

We extracted patients aged ≥65 years with nonvalvular AF in whom OACs were initiated from a universal administrative claims database incorporating primary and hospital care records in Shizuoka, Japan, between 2012 and 2018. Frailty was assessed using the electronic frailty index (eFI). The association of frailty with bleeding events and ischemic stroke/transient ischemic attack were evaluated using the Fine-Gray model and restricted cubic spline model.

**Results:**

Among 12,585 patients with AF, 7.8% were categorized as fit, 31.5% as mildly frail, 34.8% as moderately frail, and 25.9% as severely frail. The risk of bleeding was associated with a higher eFI (adjusted subdistribution hazard ratio [95% CI] for fit or mild frailty: 1.15 [1.02-1.30]; moderate frailty: 1.42 [1.24-1.61]; and severe frailty: 1.86 [1.61-2.15]), whereas the association was weaker for ischemic stroke/transient ischemic attack. The spline models demonstrated that the relative hazard for bleeding increased steeply with increasing eFI.

**Conclusion:**

Patients with frailty in whom OAC therapy is initiated have higher risk of bleeding, highlighting the importance of discussing this increased risk with patients with AF who have frailty and assessing frailty at the time of OAC initiation.

## Essentials

• The prognostic value of frailty for adverse events after initiation of oral anticoagulants (OACs) is unclear.
• The association of frailty with bleeding and embolic events was evaluated.
• Patients with frailty in whom OAC therapy is initiated have a higher risk of bleeding.
• The increased risk of bleeding in frail patients with AF should be shared with these patients.

## 1 Introduction

Atrial fibrillation (AF) is associated with a substantial risk of systemic embolic events [1,2], and the risk is particularly high in older patients with AF [2,3]. Oral anticoagulants (OACs) have been shown to reduce the rate of embolic complications, and their use is strongly supported in international clinical practice guidelines [4,5]. However, they remain underused in clinical practice, particularly for frail patients [[6], [7], [8]], despite recommendations to use OACs regardless of the frailty status [9,10].

The efficacy of OACs is assumed to be achieved at the expense of an increased risk of bleeding. Frailty is an age-related cumulative decline in physiologic systems and is known to be associated with higher risk of falls, fractures, gastrointestinal bleeding, and cerebral trauma, frequently precluding the initiation of OACs [11,12]. However, the precise association between frailty and subsequent embolic or bleeding events among patients with AF treated with OACs in the community population is unknown. Previous studies that evaluated the association of frailty with outcomes in patients with AF treated with OACs were predominantly small-scale studies or their assessments were not made in community settings [[13], [14], [15]]. Furthermore, there are gaps between knowledge from randomized controlled trials (RCTs) and that from real-world clinical practice [16] because RCTs often include patients with fewer chronic medical conditions, less frailty, and lower risk of adverse outcomes than the real-world population.

We, therefore, investigated the association between frailty and clinical outcomes among patients with AF treated with OACs in a large community-based cohort derived from administrative claims data, incorporating primary and hospital care records, in Japan.

## 2 Methods

### 2.1 Study design and data source

We conducted a cohort study using the Shizuoka Kokuho Database (SKDB) between April 2012 and September 2018 [17]. SKDB is an administrative claims database of beneficiaries in the municipal government insurance program (national health insurance and late-stage medical care system for the elderly) in Shizuoka Prefecture, Japan. All data in SKDB were anonymized. Of all residents aged <75 years, 22.3% are enrolled in the national health insurance system (eg, self-employed and unemployed), and all residents aged ≥75 years are enrolled in the late-stage medical care system for the elderly. We utilized basic information (ie, age, sex, and date of death) and health insurance claims (eg, monthly claims for patients’ diagnoses, procedures, laboratory tests ordered, drugs dispensed, and dates of hospital admissions) from SKDB. This study was approved by the Ethnic Committee of Shizuoka Graduate University of Public Health (Shizuoka, Japan) (#SGUPH_2021_001_006).

### 2.2 Study population and follow-up

We selected patients with nonvalvular AF in whom OAC therapy (warfarin or DOACs: apixaban, dabigatran, edoxaban, or rivaroxaban) was initiated between April 2013 and March 2018. We designated the month of dispensation of the first OAC as the “index month.” We included patients aged ≥65 years in whom OAC monotherapy had been initiated in an outpatient setting and who had at least 1 record of AF diagnosis (International Classification of Diseases, 10th Revision, code I48x but not the disease code for valvular AF or AF after surgery) in the preceding 12 months. We excluded patients who had been continuously enrolled in an insurance plan for <12 months (baseline period) before OAC initiation or with alternate potential indications for OAC therapy besides nonvalvular AF based on previous diagnoses of the following conditions: venous thromboembolism, rheumatic mitral valve disease, intracardiac thrombosis, mechanical or bioprosthetic heart valve, mitral valve repair, or valvular AF (Supplementary Table S1). Follow-up of outcomes started in the index month (first OAC prescription). Each patient was censored at the occurrence of the outcome of interest, death, end of enrollment in the plan, or end of the study period (September 2018), whichever came first. Embolic or bleeding events were not competing risks for each outcome of interest. For example, if bleeding occurred before an embolic event, the patient was not censored at the time of bleeding but at the time of the embolic event.

### 2.3 Patient characteristics

**Figure 1:**
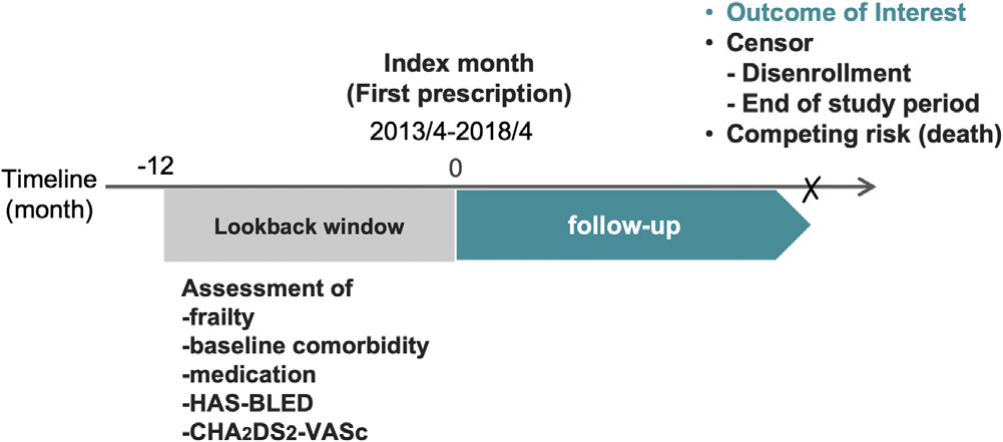
Study design timeline. CHA_2_DS_2_-VASc, congestive heart failure, hypertension, age ≥75 years, diabetes mellitus, stroke, valvular disease, age 65 to 74; HAS-BLED, age>65 years, hypertension, abnormal renal and liver function, prior stroke, bleeding history or predisposition, labile international normalized ratio, and drugs/alcohol concomitantly.

Information on the demographics (ie, sex and age) of each patient was extracted from the insurance subscriber list. We assessed the patients’ baseline comorbidities and previous medication use using recorded diagnoses and dispensation claims during the 12 months preceding the index month and in the index month (Figure 1; Supplementary Table 2A, B). We also collected data on the daily dose of DOACs in the index month among patients initiating DOAC therapy. Drug dosing was classified as reduced dose (apixaban, 2.5 mg twice a day; dabigatran, 110 mg twice a day; edoxaban, 30 mg every day; and rivaroxaban, 10 mg every day) or standard dose (apixaban, 5 mg twice a day; dabigatran, 150 mg twice a day; edoxaban, 60 mg every day; and rivaroxaban, 15 mg every day). The approved dose of rivaroxaban in Japan was 10 mg once daily for patients with a creatinine clearance rate of 15 to 49 mL/min or 15 mg once daily for patients with a creatinine clearance rate of ≥50 mL/min, based on pharmacokinetic modeling data and the results of the J-ROCKET AF trial (ie, a trial compared the safety of a Japan-specific rivaroxaban dose with warfarin) [18]. Dose data of patients in whom neither the standard nor reduced dose was initiated were treated as missing.

To determine the risk scores for bleeding and thromboembolism, we utilized the HAS-BLED and CHA_2_DS_2_-VASc scores, respectively. The scores were calculated based on diagnosis claims recorded in the index month and the previous 12 months, using the following factors: age over 65 years, hypertension, abnormal renal and liver function, prior stroke, bleeding history or predisposition, labile international normalized ratio, and drugs/alcohol concomitantly for HAS-BLED; and congestive heart failure, hypertension, age of 75 years or older, diabetes mellitus, stroke, valvular disease, age 65 to 74 years, and sex category for CHA_2_DS_2_-VASc (Supplementary Table 3A, B). The labile international normalized ratio, a component of the HAS-BLED score, was excluded from the calculation because this datum was unavailable in SKDB.

### 2.4 Frailty assessment

We evaluated the patients’ frailty using the electronic frailty index (eFI) [19]. eFI is a coding-based algorithm based on the cumulative deficit model as the theoretical framework. This algorithm was developed from electronic health records in the United Kingdom. The cumulative deficit model is an accumulation of age-related deficits: signs, symptoms, diseases, disabilities, and polypharmacy. eFI was validated for relevant outcomes (eg, mortality, nursing home admission, and frailty phenotype). Because 1 of the 36 variables included in the index (ie, falls) is not available in the Japanese claims coding system, we calculated the score based on 35 variables using the records of diagnoses, according to the International Classification of Diseases, 10th Revision, codes, and the dispensation records for the previous 12 months and the index month (Supplementary Table 4) [20]. eFI is used to categorize patients into 4 groups: fit (eFI score of 0-0.12), mildly frail (>0.12-0.24), moderately frail (>0.24-0.36), and severely frail (>0.36) [19].

### 2.5 Outcomes

The outcomes of interest were bleeding events and embolic events defined using diagnostic codes. The primary bleeding outcome was a composite outcome of major and minor bleeding. Major bleeding was defined as events including intracranial bleeding, gastrointestinal bleeding, or bleeding with shock in an inpatient setting. Minor bleeding was defined as other bleeding events that are not classified as major bleeding, recorded in an inpatient or outpatient setting (Supplementary Table 5). The embolic outcome was a secondary outcome, including ischemic stroke and transient ischemic attack (TIA), diagnosed in an inpatient setting. Major bleeding was also assessed as a secondary outcome.

### 2.6 Statistical analysis

To describe patients’ characteristics and the prevalence of the 35 deficits evaluated to calculate eFI, continuous variables are presented as medians and IQRs and categorical variables are presented as numbers and percentages. There were no missing data, except for data on dose category. To assess the associations of bleeding and thromboembolism risk scores with frailty, we tabulated and displayed the HAS-BLED and CHA_2_DS_2_-VASc scores according to the eFI groups. Spearman correlation coefficients of 2 risk scores with eFI were also estimated. We compared the frequencies of the deficits used to calculate eFI across the categories of both these scores (HAS-BLED and CHA_2_DS_2_-VASc). We assessed the percentage of patients on DOACs in whom treatment with a reduced-dose regimen was initiated according to the eFI groups.

We evaluated the outcomes using cumulative incidence functions for up to 64 months and compared the curves across eFI categories using the Gray test. Death was considered a competing risk. We evaluated the association between frailty and outcomes using univariable and multivariable Fine-Gray subdistribution hazard models after adjustment for the following covariates: sex, baseline comorbidities (cancer; chronic kidney disease; chronic obstructive pulmonary disease; depression; diabetes mellitus; heart failure; hypertension; liver disease; peptic ulcer; peripheral arterial disease; previous admission for myocardial infarction, bleeding, and stroke; rheumatoid arthritis; and sleep apnea syndrome), and the use of medications (antihypertensive drugs, antidiabetic drugs, nitrates, statins, nonsteroidal anti-inflammatory drugs, antiarrhythmic drugs, antiplatelet drugs, other lipid-lowering drugs, antidepressants, and antacids). Because our intention was to investigate whether coding-based frailty is associated with outcomes, age was not included in the adjusted model in the main analysis. However, an adjustment for age was made in a supplementary analysis. We also conducted an analysis adjusting for sex, comorbidities (cancer, chronic obstructive pulmonary disease, depression, liver disease, previous admission for bleeding, previous admission for stroke, rheumatoid arthritis, and sleep apnea syndrome), and medication use (statins, nonsteroidal anti-inflammatory drugs, other lipid-lowering drugs, and antidepressants), with the exception of eFI components that were not related to stroke.

To depict the associations between eFI as a continuous variable and the outcomes, we constructed restricted cubic spline models with 4 knots at the quintile points, adjusted for sex, baseline comorbidities, and the use of medications. We used an eFI score of 0.12, which is the threshold between fit and mild frailty, as the reference point. All statistical analyses were performed using SAS, version 9.4 (SAS Institute).

## 3 Results

### 3.1 Patient characteristics

A total of 12,585 patients with previously diagnosed AF initiating OAC therapy (median [IQR] age, 80 [72–85] years; 45.4% female) were identified (Supplementary Figure 1). In total, 980 (7.8%) were categorized as fit, 3967 (31.5%) as mildly frail, 4385 (34.8%) as moderately frail, and 3253 (25.9%) as severely frail (Table 1). Of these patients, 17.8% were prescribed warfarin, 23.6% apixaban, 10.0% dabigatran, 15.1% edoxaban, and 33.5% rivaroxaban. Apixaban was more frequently prescribed for the severely frail (27.8%) than for those with lower eFI scores. Compared with patients who were fit, patients with severe frailty were more likely to have a high bleeding risk (HAS-BLED ≥ 3: fit 36.7% vs severely frail 95.3%) or high thromboembolism risk (CHA_2_DS_2_-VASc ≥ 4: fit 35.0% vs severely frail 98.9%) (Supplementary Figure 2). The CHA_2_DS_2_-VASc score has a stronger correlation with eFI (Spearman ρ = 0.63; 95% CI, 0.62-0.64) than HAS-BLED score (Spearman ρ = 0.54; 95% CI, 0.53-0.55).

**Table 1:** Baseline characteristics of 12,585 oral anticoagulant initiators among patients with atrial fibrillation^a^.

Characteristic	eFI categories				
	Total (n = 12,585)	Fit (n = 980)	Mild (n = 3967)	Moderate (n = 4385)	Severe (n = 3253)
Follow-up (mo)	31 (17, 47)	35 (20, 49)	33 (18, 48)	32 (17, 47)	28 (15, 43)
Age (y)	80.0 (72.0, 85.0)	72.0 (69.0, 81.0)	74.0 (70.0, 83.0)	81.0 (72.0, 86.0)	83.0 (78.0, 87.0)
Age groups (y), n (%)					
65-74	4799 (38.1)	619 (63.2)	2025 (51.1)	1484 (33.8)	671 (20.6)
75-84	4222 (33.6)	227 (23.2)	1170 (29.5)	1580 (36.0)	1245 (38.3)
≥85	3564 (28.3)	134 (13.7)	772 (19.5)	1321 (30.1)	1337 (41.1)
Sex					
Male, n (%)	6872 (54.6)	678 (69.2)	2509 (63.3)	2307 (52.6)	1378 (42.4)
Female, n (%)	5713 (45.4)	302 (30.8)	1458 (36.8)	2078 (47.4)	1875 (57.6)
Type of OAC					
Warfarin, n (%)	2245 (17.8)	189 (19.3)	709 (17.9)	745 (17.0)	602 (18.5)
Apixaban, n (%)	2974 (23.6)	172 (17.6)	849 (21.4)	1050 (24.0)	903 (27.8)
Dabigatran, n (%)	1258 (10.0)	126 (12.9)	400 (10.1)	449 (10.2)	283 (8.7)
Edoxaban, n (%)	1895 (15.1)	142 (14.5)	566 (14.3)	667 (15.2)	520 (16.0)
Rivaroxaban, n (%)	4213 (33.5)	351 (35.8)	1443 (36.4)	1474 (33.6)	945 (29.1)
Baseline comorbidities					
Cancer, n (%)	4531 (36.0)	189 (19.3)	1109 (28.0)	1608 (36.7)	1625 (50.0)
Chronic kidney disease, n (%)	726 (5.8)	8 (0.8)	81 (2.0)	228 (5.2)	409 (12.6)
COPD, n (%)	1524 (12.1)	27 (2.8)	265 (6.7)	575 (13.1)	657 (20.2)
Depression, n (%)	737 (5.9)	2 (0.2)	68 (1.7)	216 (4.9)	451 (13.9)
Diabetes mellitus, n (%)	8654 (68.8)	269 (27.5)	2399 (60.5)	3252 (74.2)	2734 (84.1)
Heart failure, n (%)	8498 (67.5)	263 (26.8)	2246 (56.6)	3211 (73.2)	2778 (85.4)
Hypertension, n (%)	10304 (81.9)	580 (59.2)	2997 (75.6)	3735 (85.2)	2992 (92.0)
Liver disease, n (%)	3776 (30.0)	150 (15.3)	947 (23.9)	1419 (32.4)	1260 (38.7)
Peptic ulcer, n (%)	2977 (23.7)	46 (4.7)	554 (14.0)	1077 (24.6)	1300 (40.0)
Peripheral arterial disease, n (%)	2362 (18.8)	16 (1.6)	354 (8.9)	886 (20.2)	1106 (34.0)
Previous admission for MI, n (%)	74 (0.6)	1 (0.1)	5 (0.1)	17 (0.4)	51 (1.6)
Previous admission for bleeding, n (%)	259 (2.1)	0 (0.0)	28 (0.7)	100 (2.3)	131 (4.0)
Previous admission for stroke, n (%)	315 (2.5)	5 (0.5)	48 (1.2)	87 (2.0)	175 (5.4)
Rheumatoid arthritis, n (%)	613 (4.9)	10 (1.0)	88 (2.2)	195 (4.5)	320 (9.8)
SAS, n (%)	186 (1.5)	2 (0.2)	32 (0.8)	61 (1.4)	91 (2.8)
Medication use					
Antihypertensives, n (%)	9770 (77.6)	575 (58.7)	2908 (73.3)	3530 (80.5)	2757 (84.8)
Antidiabetic drugs, n (%)	1881 (15.0)	68 (6.9)	524 (13.2)	710 (16.2)	579 (17.8)
Nitrates, n (%)	1352 (10.7)	15 (1.5)	223 (5.6)	486 (11.1)	628 (19.3)
Statins, n (%)	3512 (27.9)	139 (14.2)	930 (23.4)	1347 (30.7)	1096 (33.7)
NSAIDs, n (%)	5390 (42.8)	205 (20.9)	1327 (33.5)	1920 (43.8)	1938 (59.6)
Antiarrhythmic drugs, n (%)	2797 (22.2)	222 (22.7)	826 (20.8)	964 (22.0)	785 (24.1)
Antiplatelet drugs, n (%)	3510 (27.9)	75 (7.7)	776 (19.6)	1309 (29.9)	1350 (41.5)
Other lipid-lowering drugs, n (%)	741 (5.9)	35 (3.6)	191 (4.8)	294 (6.7)	221 (6.8)
Antidepressants, n (%)	1136 (9.0)	11 (1.1)	163 (4.1)	396 (9.0)	566 (17.4)
Antacid, n (%)	5613 (44.6)	110 (11.2)	1153 (29.1)	2130 (48.6)	2220 (68.2)
HAS-BLED	3 (3, 4)	2 (2, 3)	3 (2, 4)	4 (3, 4)	4 (4, 5)
CHA_2_DS_2_-VASc	5 (4, 6)	3 (2, 4)	4 (4, 5)	6 (5, 6)	7 (6, 8)

CHA_2_DS_2_-VASc, congestive heart failure, hypertension, age ≥75 years, diabetes mellitus, stroke, valvular disease, age 65 to 74; COPD, chronic obstructive pulmonary disease; eFI, electronic frailty index; HAS-BLED, age>65 years, hypertension, abnormal renal and liver function, prior stroke, bleeding history or predisposition, labile international normalized ratio, and drugs/alcohol concomitantly; MI, myocardial infarction; NSAID, nonsteroidal anti-inflammatory drug; OAC, oral anticoagulant; SAS, sleep apnea syndrome.

a Values are represented as medians (Q25, Q75) for continuous variable and numbers and percentages for categorical variables.

**Figure 2:**
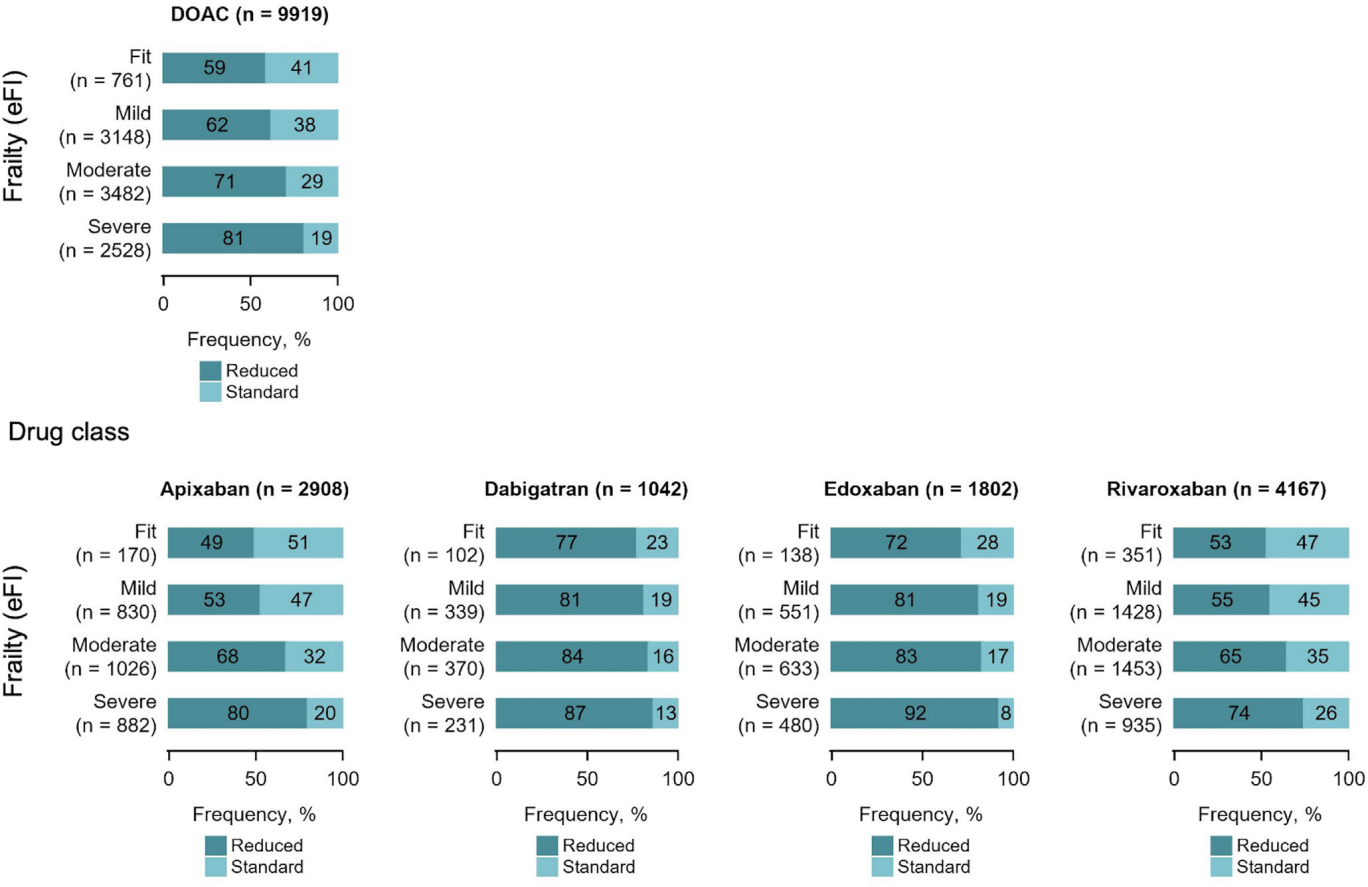
Associations between frailty and direct oral anticoagulant dose. DOAC, direct oral anticoagulant; eFI, electronic frailty index.

The frequency of each deficit used to calculate eFI was higher among those in the severely frail category (Supplementary Table 6). The prevalence of each of the 35 deficits used to calculate eFI, not only those included in the bleeding and stroke risk scores (eg, hypertension, diabetes mellitus, and cerebrovascular disease) but also frailty-specific deficits (eg, activity limitation, fragility fracture, weight loss, and anorexia), was higher in patients with higher risk scores (Supplementary Figures 3 and 4). Patients in the higher eFI categories were more likely to receive reduced-dose DOACs than the fit patients (Figure 2).

### 3.2 Incidence of clinical outcomes

During follow-up (median, 31 months; 25th-75th percentiles, 17-47 months), 5226 bleeding events and 493 ischemic stroke/TIA events occurred, yielding annual event rates of 15.5 and 1.5 per 100 patient-years, respectively (Table 2). The event rate was 1.2 per 100 patient-years for major bleeding. The overall 5-year cumulative incidence was 53.1% (95% CI, 51.6%-54.5%) for bleeding, 5.7% (4.9%-6.5%) for ischemic stroke/TIA, and 5.1% (4.4%-5.8%) for major bleeding (Supplementary Figure 5).

**Table 2:** Summary of clinical outcomes and their association with frailty.

Outcome	Events (n)	Event rate (95% CI)^a^	Unadjusted sHR (95% CI)	Adjusted sHR (95% CI)^b^
Bleeding				
Total	5226	15.5 (15.1-15.9)	-	-
Fit	318	11.2 (10.1-12.6)	Ref	Ref
Mild	1414	12.8 (12.1-13.5)	1.15 (1.02-1.30)	1.15 (1.02-1.30)
Moderate	1826	15.4 (14.7-16.1)	1.44 (1.28-1.62)	1.42 (1.24-1.61)
Severe	1668	21.0 (20.0-22.1)	1.98 (1.76-2.23)	1.86 (1.61-2.15)
Ischemic stroke/TIA				
Total	493	1.5 (1.3-1.6)	-	-
Fit	28	1.0 (0.7-1.4)	Ref	Ref
Mild	155	1.4 (1.2-1.6)	1.40 (0.94-2.09)	1.27 (0.83-1.94)
Moderate	175	1.5 (1.3-1.7)	1.43 (0.96-2.13)	1.25 (0.80-1.97)
Severe	135	1.7 (1.4-2.0)	1.55 (1.03-2.32)	1.26 (0.77-2.06)
Major bleeding				
Total	419	1.2 (1.1-1.4)	-	-
Fit	21	0.7 (0.5-1.1)	Ref	Ref
Mild	114	1.0 (0.9-1.2)	1.37 (0.86-2.18)	1.30 (0.81-2.09)
Moderate	161	1.4 (1.2-1.6)	1.76 (1.12-2.77)	1.61 (0.98-2.63)
Severe	123	1.6 (1.3-1.8)	1.87 (1.18-2.97)	1.61 (0.93-2.80)
Competing event (all-cause death)				
Total	2027	6.0 (5.8-6.3)	-	-
Fit	94	3.3 (2.7-4.1)	-	-
Mild	453	4.1 (3.7-4.5)	-	-
Moderate	711	6.0 (5.6-6.4)	-	-
Severe	769	9.7 (9.0-10.4)	-	-

Ref, reference; sHR, subdistribution hazard ratio; TIA, transient ischemic attack.

a Event rate per 100 patient-years.

b Models adjusted for sex, medical history, and medications.

**Figure 3:**
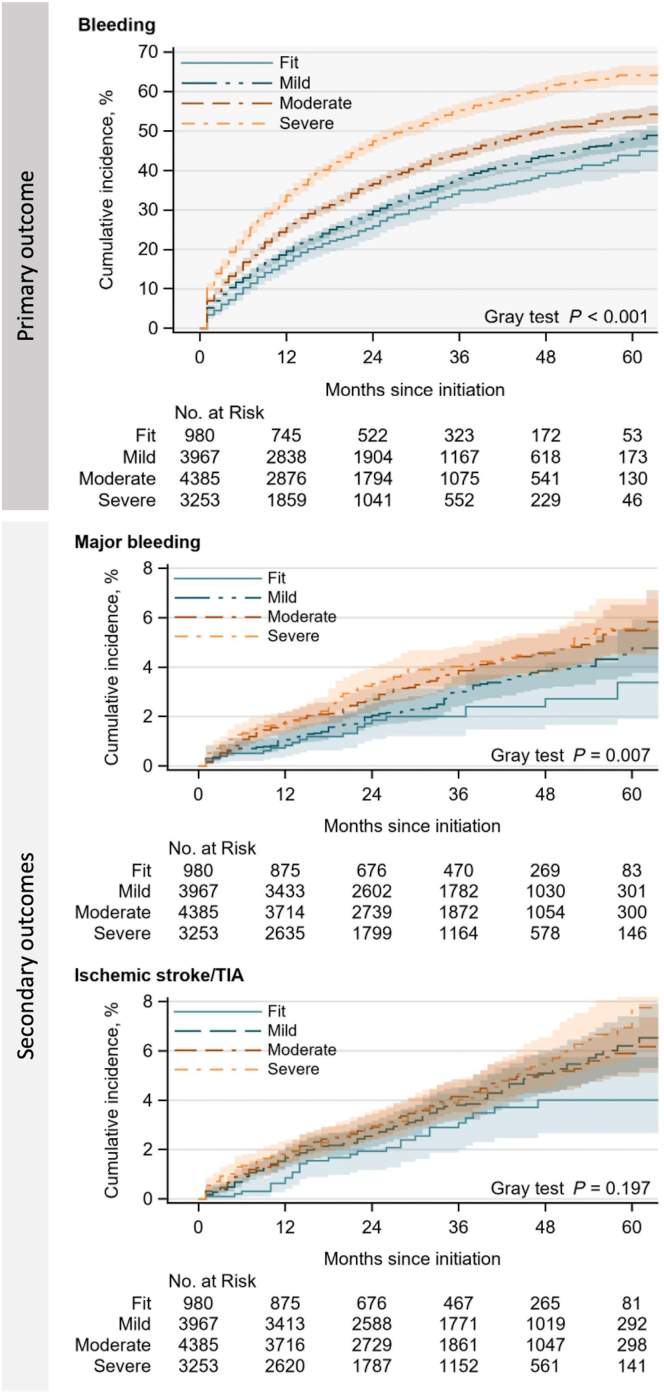
Cumulative incidence of outcomes stratified by electronic frailty index categories. *Note:* Shaded bands indicated 95% CIs. The y-axis ranges from 0 to 70%, indicating the cumulative incidence of bleeding, and from 0 to 8% indicating the cumulative incidence of ischemic stroke/transient ischemic attack and major bleeding. TIA, transient ischemic attack.

The annual event rates among patients who were fit and those who were severely frail were 11.2 and 21.0 per 100 patient-years for bleeding, respectively, 1.0 and 1.7 per 100 patient-years for ischemic stroke/TIA, respectively, and 0.7 and 1.5 per 100 patient-years for major bleeding, respectively (Table 2). Figure 3 shows the cumulative incidence functions for the primary and secondary bleeding outcomes compared across the frailty categories. The cumulative incidence curves for the frailty categories were substantially separated for the bleeding end points but minimally different for ischemic stroke/TIA. For major bleeding, the cumulative incidence curves for the moderately and severely frail patients differed only slightly but differed considerably from the curve for the fit patients.

### 3.3 Associations between frailty and clinical outcomes

**Figure 4:**
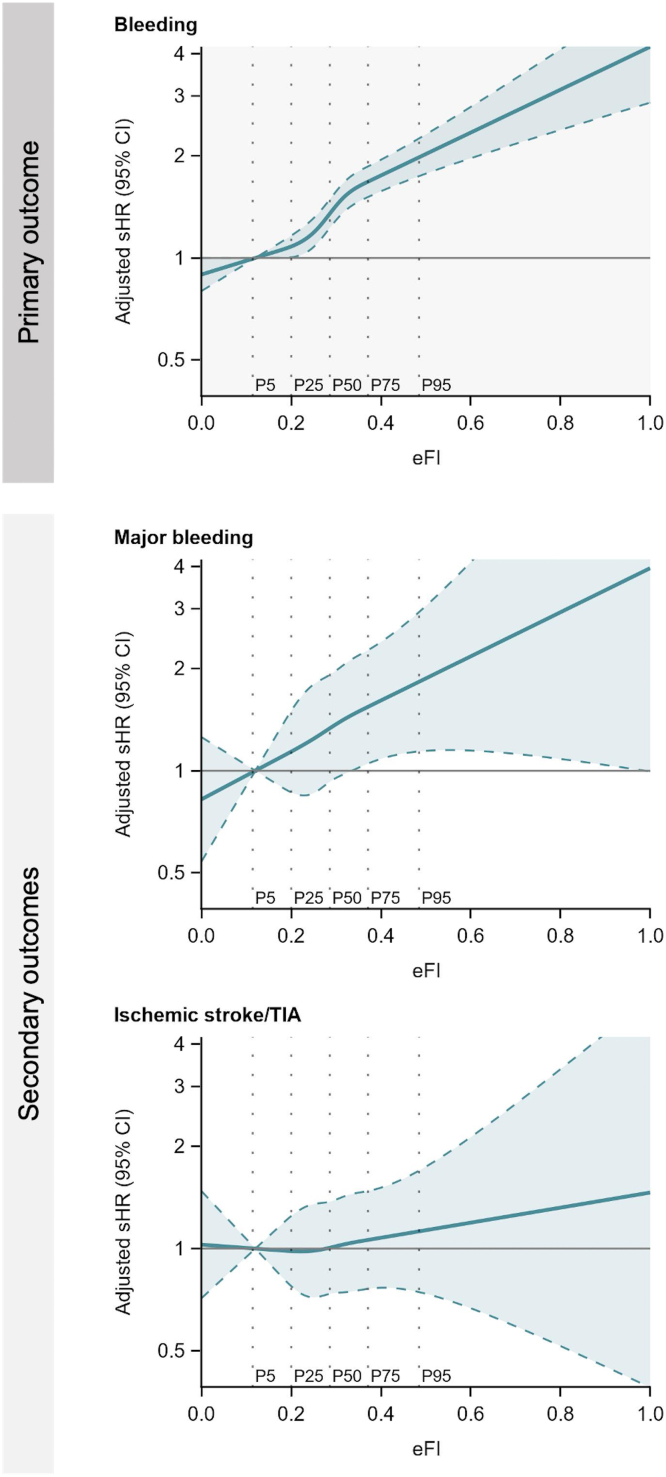
Associations between electronic frailty index and outcomes (eFI). *Note:* The model was adjusted for sex, medical history, and medications. Subdistribution hazard ratios with 95% CIs are plotted. An eFI score of 0.12 (cutoff score between fit and mild frailty) was the reference standard. Bleeding events were defined as any outpatient or inpatient bleeding event. The vertical dotted lines show thresholds for the percentiles of eFI value. eFI, electronic frailty index; sHR, subdistribution hazard ratio; TIA, transient ischemic attack.

After adjustment for sex, baseline comorbidities, and medications, the risk of bleeding was associated with increased eFI scores, with adjusted subdistribution hazard ratios (sHRs) of 1.15 (95% CI, 1.02-1.30) in the patients with mild frailty, 1.42 (95% CI, 1.24-1.61) in those with moderate frailty, and 1.86 (95% CI, 1.61-2.15) in those with severe frailty compared with that in the fit patients. On the other hand, the association of frailty was weaker for ischemic stroke/TIA (adjusted sHR [95% CI] for fit or mild frailty: 1.27 [0.83-1.94]; moderate frailty: 1.25 [0.80-1.97]; and severe frailty: 1.26 [0.77-2.06]) (Table 2). For the secondary bleeding outcome, we observed an upward trend in sHR with increased eFI severity (adjusted sHR [95% CI]: mild frailty, 1.30 [0.81-2.09]; moderate frailty, 1.61 [0.98-2.63]; and severe frailty, 1.61 [0.93-2.80]). An adjusted model, including age, showed similar results, except for major bleeding (Supplementary Table 7). When not adjusted for age and indicators of eFI, frailty was associated with ischemic stroke/TIA (Supplementary Table 8). The spline curves were consistent with these results: patients with lower eFI scores had a lower relative subdistribution hazard for bleeding, and the relative subdistribution hazard increased steeply with increasing eFI (Figure 4). After stratification for warfarin or DOACs, the relative risk of bleeding in the severely frail patients showed similar patterns in patients treated with either DOACs or warfarin (Supplementary Figure 6).

## 4 Discussion

In this contemporary population-based study of older patients with AF aged ≥65 years who had initiated OAC therapy, the prevalence of frailty was common in the community setting, and 26% of the subjects were severely frail. We found a strong association between frailty and increased risk of bleeding. Our results suggest that the increased risk of bleeding in frail patients with AF should be shared with these patients.

Overall, 26% of patients with AF aged ≥65 years on OACs were severely frail, whereas 7.8% were fit. A systematic review reported that the prevalence of frailty among patients with AF was 50.4% to 75.4%, depending on the age distribution of the target and how frailty was defined [18]. A self-reported frailty scale and other frailty indices were included in these definitions, but no frailty measure based on health care data was reported. However, the distribution of eFI and the median age in our study were similar to those in a previous study that included patients with AF in a primary care setting (median age, 80 years; severe frailty, 23%; and fit, 10.5%) [12].

We identified a considerable association between frailty and increased risk of bleeding. A higher risk of bleeding among frail patients with AF has been a concern when initiating OAC [21,22], and previous studies have reported a positive association between frailty and bleeding among patients with AF. This association has been most evident in observational, real-world studies [12,13,22,23]. Single-center studies from Italy [13] and Japan [22] as well as a post hoc analysis from an RCT [15] found that frailty was associated with major bleeding, defined based on International Society on Thrombosis and Hemostasis criteria. Although large-scale and community-setting studies were limited, a study from the United Kingdom using primary care records reported the association between frailty and gastrointestinal bleeding [12]. Although the definitions of frailty and bleeding have variations, the result of the association between frailty and bleeding in the present study is consistent with those in previous studies. Several observational studies have reported that age is an independent predictor of stroke but not of bleeding in older patients with AF [24,25]. Our observation of an association between frailty and bleeding indicates that frailty should be considered a risk factor for bleeding, as great as or greater than chronologic age. Although “falls,” which is also included as a predictor in eFI (but was not available in the present study), is a component of frailty, a prospective cohort study found that a high risk of falls was not associated with major bleeding [26]. Frailty, a cumulative decline in physiologic systems, may contribute to bleeding rather than the high risk of fall itself.

Importantly, our study does not support the withholding or withdrawal of OAC initiation because of a high risk of bleeding. The updated European Society of Cardiology (ESC) clinical practice guidelines (2020 European Society of Cardiology Guidelines for the diagnosis and management of AF developed in collaboration with the European Association for Cardio-Thoracic Surgery) [5] state that “frailty, comorbidities, and increased risk of falls do not outweigh the benefits of OAC given the small absolute risk of bleeding in anticoagulated elderly patients” because the net clinical benefit of OACs is considered to be much greater than any potential harm. However, to date, the precise relationship (or formal quantitative analysis) between frailty status and clinical outcomes (bleeding and stroke) in patients with AF with an indication of OACs remains largely unclear [27]. There is uncertainty about the balance between benefit (ie, stroke prevention) and harm (ie, bleeding events) among patients with frailty.

The crude incidence of bleeding events is considerably high in frail patients, and a large-scale study including patients with AF with a broad range of clinical backgrounds is warranted. A recent small-scale RCT showed that more bleeding events (ie, major or clinically relevant nonmajor bleeding) occurred in frail patients than in nonfrail patients (21.4% [42/185] vs 15.6% [53/289], respectively) [28]. Because the safety and efficacy of OACs for very frail patients with AF remain controversial, our study suggests that trials are required to assess the safety and efficacy of withholding OACs after bleeding events in frail elderly patients [24,25,29]. Moreover, frailty should be incorporated in risk assessment as part of shared decision making for OAC initiation. The high bleeding risk in frail patients must be recognized.

In the present study, we found that patients with moderate-to-severe frailty had a limited increase in the incidence of ischemic stroke/TIA compared with those who were not frail after OAC initiation. The association between frailty and stroke is controversial [23,29]. An unclear association between frailty and increased risk of stroke in patients with AF has predominantly been reported in studies conducted in primary care settings [12,30]. Other studies that have reported an association between frailty and stroke in patients with AF were prospective cohort studies or post hoc analyses from RCTs[12,15,24,25,31,32]. In our study of patients with AF aged ≥65 years (median age, 80 years), frailty was not strongly associated with ischemic stroke/TIA after adjustment for sex, comorbidities, and medication use. However, when indicators of eFI were not included in the adjustments, frailty was found to be associated with ischemic stroke/TIA. This suggests that the association between frailty and stroke events was weakened by adjusting for comorbidities and concomitant medication use, which are also included in eFI.

In this study, which was based on a contemporary claims database, DOACs were more frequently prescribed (n = 10,340; 82%) than warfarin and more frequently prescribed than in previous studies [12,15,16,22,31,32]. A recent meta-analysis of RCTs and an observational study showed that DOACs were preferable over warfarin in terms of the risk of bleeding [4,16,33]. In the United States clinical practice guidelines [4], DOACs were strongly recommended as first-line therapy in preference to warfarin among eligible patients [4,5]. The proportion of DOAC prescriptions has increased rapidly since the entry of DOACs into the market in Japan, the United Kingdom, and the United States [[34], [35], [36], [37]]. The current prescription trends must be taken into consideration when interpreting our findings.

### 4.1 Limitations

This study should be interpreted in the context of several limitations. First, the validity of disease coding in administrative claims databases has been reported to have suboptimal accuracy. However, in a previous study, we found that the eFI calculated from Japanese claims accurately predicted mortality and the use of long-term care services. Second, we evaluated the association between frailty of patients with AF initiating OAC treatment and outcomes, whereas most previous studies have described the associations between frailty and clinical outcomes among patients with AF with or without OAC treatment. Therefore, patients with relatively more severe AF might have been included in this study (99.3% of patients had CHA_2_DS_2_-VASc scores of ≥2) than in previous research. Third, we included specific disease codes based on our clinical knowledge of the events (stroke/TIA and bleeding). The codes selected for the outcome were reviewed by experts. However, the definitions have not been validated. These circumstances may have caused misclassification. In particular, clinical information on bleeding, such as drop in hemoglobin levels, was not available in the claims data. Therefore, we could not define the bleeding events based on standardized criteria (eg, criteria defined by the International Society on Thrombosis and Haemostasis). Fourth, although our study was conducted in Japan, where the majority of patients are of Asian ethnicity, it is important to acknowledge that we did not collect specific data on race or ethnicity. Although this limits our ability to draw conclusions about how race or ethnicity might impact the outcomes we studied, it is worth noting that the homogeneity of our study population may limit generalizability to other populations with different demographic characteristics.

## 6 Conclusion

Increased severity of frailty is associated with a higher risk of bleeding. The increased risk of bleeding in frail patients with AF should be shared with these patients, and personalized management strategies should be implemented to minimize the bleeding risk.
