# Supplementary material for: Frailty and subsequent adverse outcomes in older patients with atrial fibrillation treated with oral anticoagulants: The Shizuoka study
Source: Res Pract Thromb Haemost. 2023 Mar 25;7(3):100129. doi: 10.1016/j.rpth.2023.100129 (PMC10165150; doi:10.1016/j.rpth.2023.100129)
Supplement: Supplementary Data [file mmc1.docx]

**Supplementary Data**

Supplement to:

Frailty and subsequent adverse outcomes in older patients with atrial fibrillation treated with oral anticoagulants: The Shizuoka study

**Supplemental Tables**

**Supplementary Table 1**. List of diagnoses for exclusion

**Supplementary Table 2A.** List of ICD-10 codes and definitions for baseline comorbidities

**Supplementary Table 2B.** List of WHO Anatomical Therapeutic Chemical (ATC) Classification codes for definitions of baseline medication use

**Supplementary Table 3A.** List of ICD-10 codes and definitions for HAS-BLED risk factors

**Supplementary Table 3B.** List of ICD-10 codes and definitions for CHA_2_DS_2_-VASc risk factors

**Supplementary Table 4.** List of ICD-10 codes and the definitions corresponding to variables in eFI

**Supplementary Table 5.** List of IC-10 codes for outcomes

**Supplementary Table 6.** Prevalence of deficits in Electronic Frailty Index

**Supplementary Table 7.** Association between frailty and outcomes after adjustment for sex, baseline comorbidities and medication use

**Supplementary Table 8.** Association between frailty and outcomes after adjustment for sex, baseline comorbidities and medication use with the exception of eFI components that were not related to stroke admission.

**Supplemental Figures**

**Supplementary Figure. 1** Cohort flow chart

**Supplementary Figure. 2** HAS-BLED and CHA_2_DS_2_-VASc scores by eFI categories

**Supplementary Figure. 3** Prevalence of deficits in eFI by HAS-BLED score group

**Supplementary Figure. 4** Prevalence of deficits in eFI by CHA_2_DS_2_-VASc score group

**Supplementary Figure. 5** Five-year cumulative incidence of outcomes by subgroup

**Supplementary Figure. 6** Associations between eFI and bleeding stratified by warfarin (n=2245) or DOAC (n= 10,340)


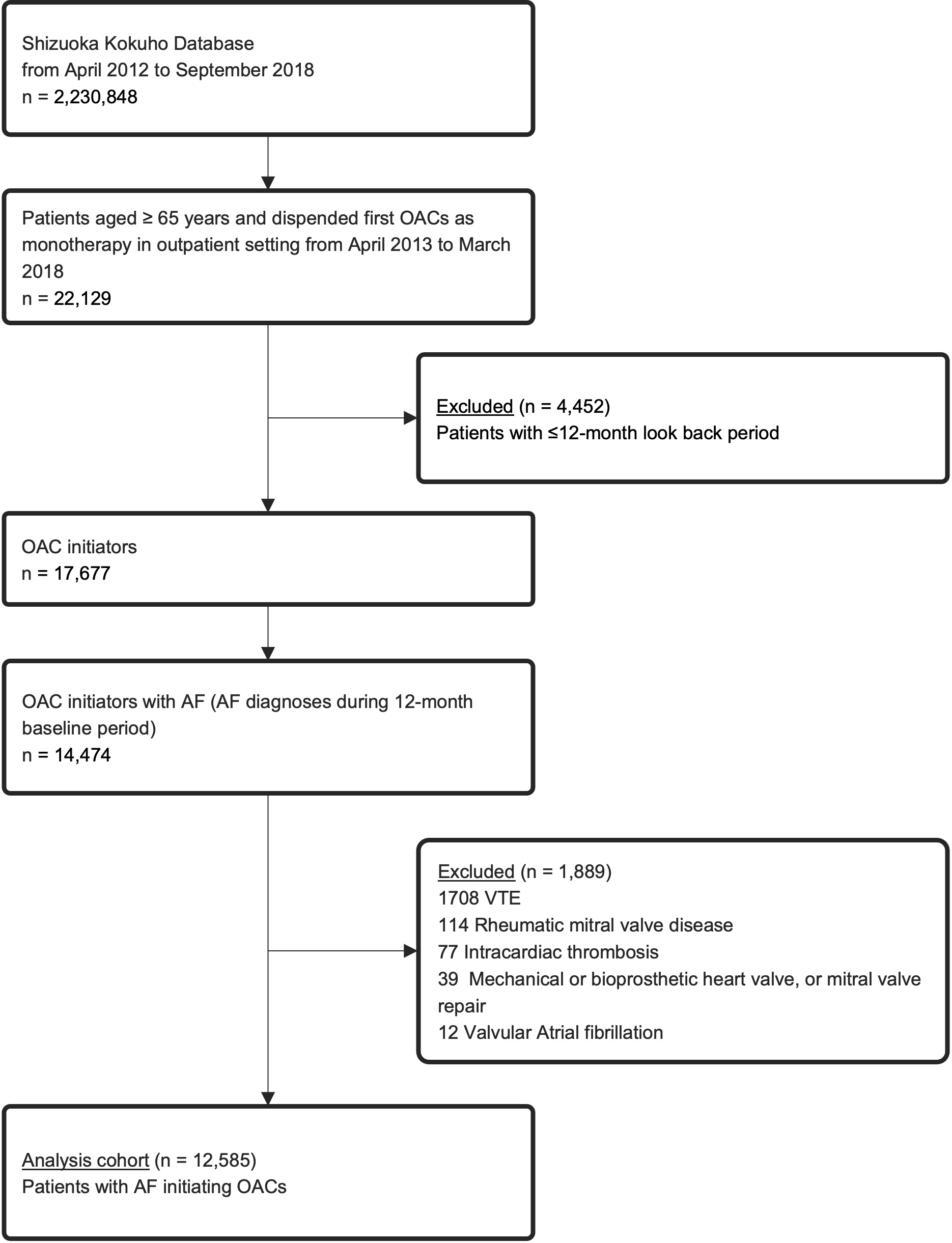


**Supplementary Figure 1.** Cohort flow chart

**Supplementary Table 1.** List of diagnoses for exclusion

| **Excluded diagnoses** | **ICD 10 code/definition** |
| --- | --- |
| Venous thromboembolism | I260, I269, I800, I801, I802, I803, I808, I809, I81, I820, I821, I822, I823, I828, I829, O223, O871, O882 |
| Rheumatic mitral valve disease | I050, I051, I052, I058, I059 |
| Intracardiac thrombosis | I513 |
| Mechanical or bioprosthetic heart valve, or mitral valve repair | Z952, Z954 |
| Valvular Atrial fibrillation | Disease code for valvular atrial fibrillation and atrial fibrillation after surgery I489 |

**Supplementary Table 2A.** List of ICD-10 codes and definitions for baseline comorbidities

| **Comorbidity** | **International Classification of Diseases (ICD) codes** |
| --- | --- |
| Cancer | Cxx |
| Chronic kidney disease | N181, N182, N183, N184, N185, N189 |
| Chronic obstructive pulmonary disease | J42, J430, J431, J432, J439, J440, J441, J448, J449 |
| Depression | F320, F321, F322, F323, F328, F329, F330, F331, F332, F333, F334, F339 |
| Diabetes mellitus | E10, E100, E101, E102, E103, E104, E105, E106, E107, E109, E11, E110, E111, E113, E114, E115, E116, E117, E119, E13, E133, E131, E130, E134, E132, E137, E136, E139, E135, E14, E140, E141, E142, E143, E144, E145, E146, E149 |
| Heart failure | I500, I501, I509 |
| Hypertension | I10 |
| Liver disease | K700, K701, K702, K703, K704, K709, K732, K739, K730, K738, K746, K741, K740, K743, K744, K745, K759, K750, K753, K754, K758, K752, K751, K768, K761, K769, K763, K765, K767, K764, K766, K760, K762 |
| Peptic ulcer disease | K259, K257, K255, K253, K251, K250, K252, K254, K256, K269, K260, K262, K263, K261, K267, K264, K266, K265, K279, K277, K270, K284, K285, K289, K287 |
| Peripheral arterial disease | I700, I701, I702, I7020, I7021, I708, I709, I739 |
| Previous admission for myocardial infarction | I210, I211, I212, I213, I214, I219, I220, I221, I228, I229 |
| Previous admission for bleeding | A162, A16, B30, D50, D62, D66, D68, D69, E07, E27, G36, G95, G96, H05, H11, H16, H20, H210, H31, H35, H40, H43, H44, H47, H60, H66, H73, H92, I21, I23, I31, I60, I61, I62, I63, I69, I78, I84, I85, I864, J04, J33, J90, J94, J95, K04, K0, K09, K12, K13, K14, K22, K25, K26, K27, K28, K29, K57, K62, K66, K76, K8, K92, L50, M25, N02, N28, N30, N32, N36, N42, N48, N50, N64, N83, N8, N89, N90, N92, N93, N939, N95, O71, O90, R04, R18, R19, R23, R31, R57, R58, S00, S01, S05, S06, S09, S10, S141, S24, S27, S30, S34, S36, S37, S39, S40, S50, S60, S70, S80, S90, T00, T06, T090, T09, T14, T79, T810, T81, T87, T90 |
| Previous admission for stroke | I63, I630, I631, I632, I633, I634, I635, I636, I638, I639 |
| Rheumatoid arthritis | M0690, M0691, M0692, M0693, M0694, M0695, M0696, M0697, M0698 |
| Sleep apnoea syndrome | G473 |

**Supplementary Table 2B.** List of WHO Anatomical Therapeutic Chemical (ATC) Classification codes for definitions of baseline medication use

| **Medication** | **WHO ATC Classification codes** |
| --- | --- |
| Antihypertensive drugs | C07, C08, C09A, C09D |
| Antidiabetic drugs | A10 |
| Nitrates | C01DA |
| Statins | C10AA |
| NSAIDs | M01A |
| Antiarrhythmic drugs | C01B |
| Antiplatelet drugs | B01AC04, B01AC22, B01AC05, B01AC06, B01AC07, B01AC23, B01AC24 |
| Other lipid-lowering drugs | C10 (except for C10AA) |
| Antidepressants | N06 |
| Antacid | A02BA, A02BC |

**Supplementary Table 3A.** List of ICD-10 codes and definitions for HAS-BLED risk factors

| **HAS-BLED risk factor** | **ICD-10 codes/definitions** |
| --- | --- |
| Hypertension | I10, I119, I120, I129, I150, I151, I152, I158, I159 |
| Abnormal renal/liver function | K702, K703, K704, K709, K730, K732, K738, K739, K740, K741, K743, K744, K745, K746, K750, K751, K752, K753, K754, K758, K759, K760, K761, K762, K763, K764, K765, K766, K767, K768, K769, N181, N182, N183, N184, N185, N189, N19, N26 |
| Stroke | I60, I600, I601, I602, I603, I604, I605, I606, I607, I608, I609, I61, I610, I611, I613, I614, I615, I616, I618, I619, I630, I631, I632, I633, I634, I635, I636, I638, I639, I64, I740, I741, I742, I743, I744, I745, I748, I749, G459 |
| Bleeding history or predisposition | D640, D641, D649, D648, D644, D643, D642, D551, D552, D550, D538, D510, D530, D560, D582, D518, D539, D580, D511, D564, D581, D520, D591, D532, D589, D571, D572, D573, D596, D594, D531, D561, D529, D588, D559, D599, D513, D569, D563, D500, D508, D528, D578, D509, D562, D512, D593, D519, D501, D570, D595, D592, D590, D521, D62, A162, A16, B30, D50, D62, D66, D68, D69, E07, E27, G36, G95, G96, H05, H11, H16, H20, H210, H31, H35, H40, H43, H44, H47, H60, H66, H73, H92, I21, I23, I31, I60, I61, I62, I63, I69, I78, I84, I85, I864, J04, J33, J90, J94, J95, K04, K0, K09, K12, K13, K14, K22, K25, K26, K27, K28, K29, K57, K62, K66, K76, K8, K92, L50, M25, N02, N28, N30, N32, N36, N42, N48, N50, N64, N83, N8, N89, N90, N92, N93, N939, N95, O71, O90, R04, R18, R19, R23, R31, R57, R58, S00, S01, S05, S06, S09, S10, S141, S24, S27, S30, S34, S36, S37, S39, S40, S50, S60, S70, S80, S90, T00, T06, T090, T09, T14, T79, T810, T81, T87, T90 |
| Labile international normalized ratio | Not applicable |
| Elderly (≥65 years) | - |
| Drugs or excessive alcohol drinking | K700, K701, F102, F105, F106, F107, F100, F101, F103, F104, T513, T519, T512, T510, T511 |

**Supplementary Table 3B.** List of ICD-10 codes and definitions for CHA_2_DS_2_-VASc risk factors

| **CHA2DS-VASc risk factor** | **ICD-10 codes/definitions** |
| --- | --- |
| Congestive heart failure | I500, I501, I509, I110 |
| Hypertension | I119, I120, I129, I150, I151, I152, I158, I159 |
|  | Antihypertensive medications listed in Supplemental Table 5B |
| Age ≥ 75 years | - |
| Diabetes mellitus | E10, E100, E101, E102, E103, E104, E105, E106, E107, E109, E11, E110, E111, E113, E114, E115, E116, E117, E119, E13, E130, E131, E132, E133, E134, E135, E136, E137, E139, E14, E140, E141, E142, E143, E144, E145, E146, E149 |
|  | Anti-diabetic medications listed in Supplemental Table 5B |
| Stroke/TIA/TE | I60, I600, I601, I602, I603, I604, I605, I606, I607, I608, I609, I61, I610, I611, I613, I614, I615, I616, I618, I619, I630, I631, I632, I633, I634, I635, I636, I638, I639, I64, I740, I741, I742, I743, I744, I745, I748, I749, G459 |
| Vascular disease | E105, E115, E135, E145, I210, I211, I212, I213, I214, I219, I220, I221, I228, I229, I420, I421, I422, I423, I424, I425, I426, I427, I428, I429, I700, I701, I702, I7020, I7021, I708, I709, I739 |
| Age 65–74 years | - |
| Sex category (female) | - |

**Supplementary Table 4.** List of ICD-10 codes and the definitions corresponding to variables in eFI

| **No.** | **eFI** | **ICD 10 code/definition** |
| --- | --- | --- |
| 1 | Activity limitation | R26, S78, S88, S98, T13.6, Y83, Z99.3, G11, G81, G82, G83, M62 |
| 2 | Anaemia and haematinic deficiency | D50, D51, D52, D53, D64 |
| 3 | Arthritis | M05, M06, M07, M09, M10, M11, M12, M13, M15, M16, M17, M18, M19, M31.5, M32, M33, M34, M35, M36 |
| 4 | Atrial fibrillation | I44, I48, I49 |
| 5 | Cerebrovascular disease | G45, G46, I6, H34 |
| 6 | Chronic kidney disease | I12, I13, N01, N03, N05, N07, N08, N18, N19, N25, I77 |
| 7 | Diabetes | E10.9, E11.9, E12.9, E13.9, E14.9 |
| 8 | Dizziness | I95, R55, R42, E86, H81, H82, H83 |
| 9 | Dyspnoea | R06 |
| 10 | Falls | Not available |
| 11 | Foot problems | B353, G575, G576, L60, M201, M202, M203, M204, M205, M206, M213, M214, M215, M216, M722, M766, M773, M775, S90, S91, S92, S93, S94, S96, S97, S99, Q66 |
| 12 | Fragility fracture | M484, S22, S32, S33, S42, S43, S62, S72, S73, M485, M800, M808, M843, M847, S02, S12, S52, S82, S92 |
| 13 | Hearing impairment | H833, H90, H91, G960, H60, H61, H62, H71, H73, H74, H92, H93 |
| 14 | Heart failure | I11, I13, I26.0, I27, I42, I43, I50, I51, I09.9, I255 |
| 15 | Heart valve disease | I05, I06, I07, I08, I34, I35, I36, I37, I390, I391, I392, I393, I394, A520, I091, I098, I38, Q230, Q231, Q232, Q233 |
| 16 | Housebound | R40, Z50, Z74, Z75.5 |
| 17 | Hypertension | I10, I11, I12, I13, H350 |
| 18 | Hypotension/syncope | I95, R55, R42, E86 |
| 19 | Ischaemic heart disease | I20, I21, I22, I23, I24, I25 |
| 20 | Memory and cognitive problems | F00, F01, F02, F03, F04, F05, F06.7, G30, G31, R41, R54, F2, F3, F41, R44, R45 |
| 21 | Mobility and transfer problems | R26, S78, S88, S98, T136, G11, G81, G82, G83, M62 |
| 22 | Osteoporosis | M80, M81, M82 |
| 23 | Parkinsonism and tremor | G122, G20, G21, G22, G23, G25, G26, G32, G35, R25 |
| 24 | Peptic ulcer | K21, K25, K26, K27, K28, K29, R12 |
| 25 | Peripheral vascular disease | I65, I70, I71, I72, I73, I771, K551, K558, K559, R02, Z958, Z959, I790, I792, Z958 |
| 26 | Polypharmacy | ≥5 drugs prescriptions for a total of ≥6 months during the baseline period |
| 27 | Requirement for care | R40 |
| 28 | Respiratory disease | J45, J46, J40, J41, J42, J43, J44, J47, J60, J61, J62, J63, J64, J65, J67, J684, J70, J13, J14, J15, J16, J18, J22, J20, J90, J961, J980 |
| 29 | Skin ulcer | I83, I98, L03, L08, L89, L97, L984 |
| 30 | Sleep disturbance | G47, F51 |
| 31 | Social vulnerability | F1, R460, R468, Z59, Z60, Z63, Z73 |
| 32 | Thyroid disease | E00, E01, E03, E04, E05, E06, E079, E890, R946 |
| 33 | Urinary incontinence | N31, N393, N394, R15, R32, Z466 |
| 34 | Urinary system disease | N30, N34, N39.0, N39.8, N39.9, R31, R33, T835 |
| 35 | Visual impairment | H25, H28, H35, H40, H43, H53, H54 |
| 36 | Weight loss and anorexia | E41, E43, E44, E46, E53, E55, E66, E83, E87, R53, R628, R63, R64, F500, F501, F508, F509 |

**Supplementary Table 5.** List of ICD-10 codes for outcomes

| **Outcomes** | | **International Classification of Diseases (ICD) codes** |
| --- | --- | --- |
| **Ischaemic stroke/TIA** | |  |
|  | Ischaemic stroke | I633, I634, I635, I636, I638, I639, I64 |
|  | TIA | G450, G451, G453, G454, G458, G459 |
|  | |  |
| **Bleeding (major and minor)** | | A162, A165, B303, D500, D62, D66, D683, D698, D699, E078, E274, G361, G951, G968, H052, H113, H168, H208, H210, H313, H350, H356, H357, H405, H431, H448, H470, H603, H669, H738, H922, I213, I230, I312, I600, I601, I602, I603, I604, I605, I606, I607, I608, I609, I610, I611, I613, I614, I615, I618, I619, I620, I621, I629, I638, I690, I691, I780, I788, I850, I864, J041, J339, J90, J942, J950, K049, K068, K092, K121, K137, K148, K226, K228, K250, K252, K254, K256, K260, K262, K264, K266, K270, K284, K290, K571, K573, K625, K649, K661, K762, K768, K859, K920, K921, K922, L508, M2506, M2509, N029, N288, N300, N304, N309, N328, N368, N421, N488, N501, N645, N830, N831, N836, N837, N838, N898, N908, N921, N922, N923, N924, N930, N938, N939, N950, O717, O901, O902, R040, R041, R042, R048, R049, R18, R195, R233, R31, R571, R58, S000, S001, S002, S003, S004, S005, S007, S008, S013, S019, S050, S051, S063, S0630, S0631, S064, S0640, S0641, S065, S0650, S0651, S066, S0660, S0661, S068, S0680, S0681, S098, S100, S101, S141, S241, S271, S2710, S2711, S272, S2720, S2721, S278, S2780, S2781, S279, S2790, S2791, S301, S302, S341, S361, S3610, S3611, S3680, S369, S3690, S3691, S370, S3700, S3701, S3780, S390, S400, S408, S500, S501, S600, S601, S701, S800, S801, S902, T009, T060, T090, T093, T140, T144, T145, T146, T794, T810, T811, T876, T905 |
| **Major bleeding** | |  |
|  | Intracranial bleeding |  |
|  | - Subarachnoid haemorrhage | I600, I601, I602, I603, I604, I605, I606, I607, I608, I609 |
|  | - Intracerebral haemorrhage | I610, I611, I613, I614, I615, I618, I619 |
|  | - Subdural haemorrhage (acute, nontraumatic) | I620 |
|  | - Nontraumatic extradural haemorrhage | I621 |
|  | - Intracranial haemorrhage (nontraumatic), unspecified | I629 |
|  | - Other cerebral infarction | I638 |
|  | - Sequelae of cerebrovascular disease | I690, I691 |
|  | - Epidural haemorrhage | S064, S0640 |
|  | - Traumatic subdural haemorrhage | S065, S0650, S0651 |
|  | Gastrointestinal bleeding/bleeding with shock |  |
|  | - Gastro-oesophageal laceration-haemorrhage syndrome | K226 |
|  | - Other specified diseases of oesophagus | K228 |
|  | - Gastric ulcer | K250, K252, K254 |
|  | - Duodenal ulcer | K260, K262, K264, K266 |
|  | - Acute haemorrhagic gastritis | K290 |
|  | - Diverticular disease of large intestine without perforation or abscess | K573 |
|  | - Gastrointestinal haemorrhage, unspecified | K922 |
|  | - Haemorrhagic shock | R571 |


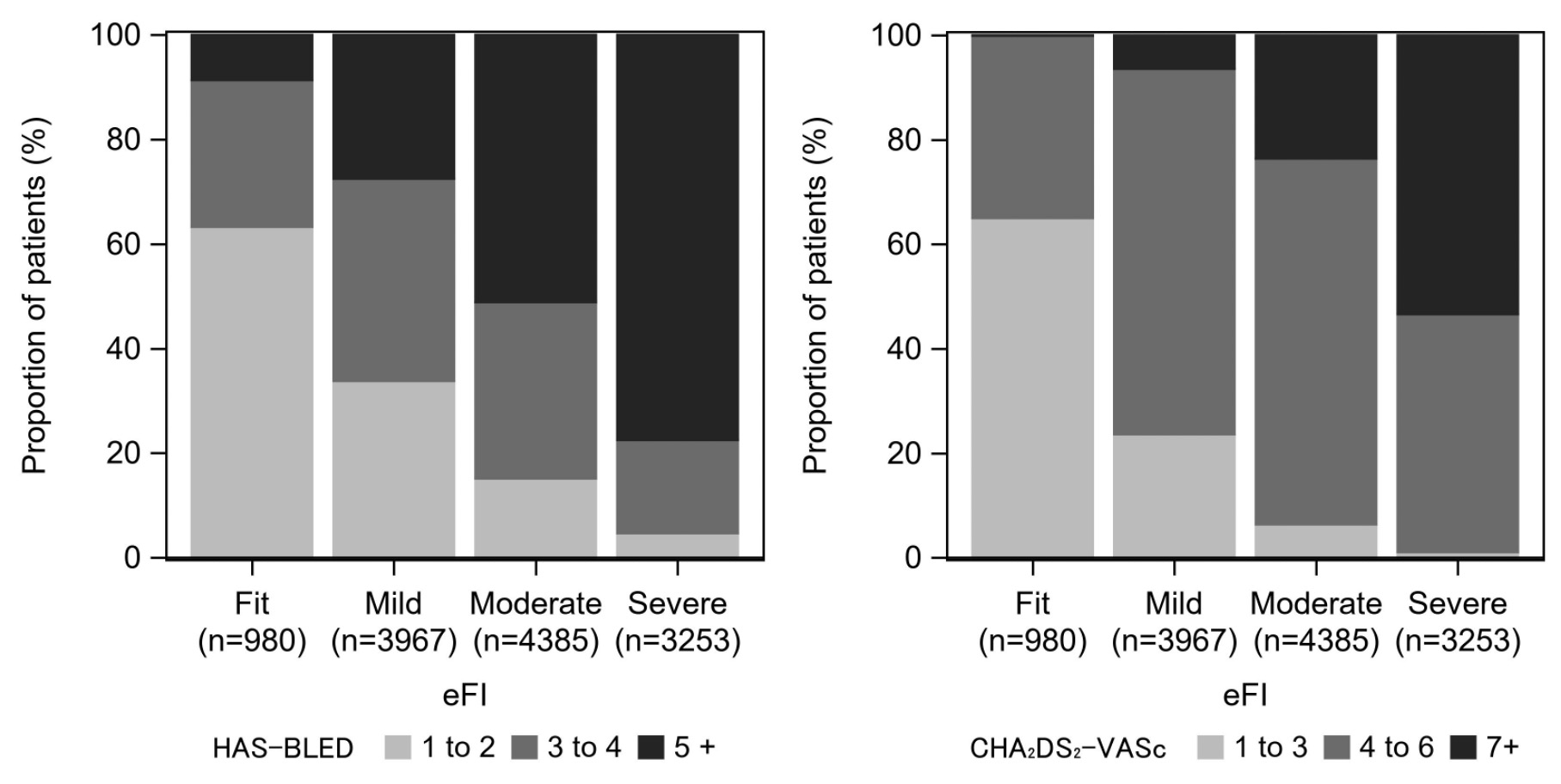


**Supplementary Figure 2.** HAS-BLED and CHA_2_DS_2_-VASc scores by eFI categories

**Supplementary Table 6.** Prevalence of deficits in Electronic Frailty Index

| **eFI (Deficits)** | **Total** | **eFI, category** | | | |
| --- | --- | --- | --- | --- | --- |
|  |  | **Fit** | **Mild** | **Moderate** | **Severe** |
|  | n=12585 | n=980 | n=3967 | n=4385 | n=3253 |
| Activity limitation | 1016 (8.1) | 0 (0.0) | 75 (1.9) | 300 (6.8) | 641 (19.7) |
| Anaemia and haematinic deficiency | 2933 (23.3) | 27 (2.8) | 473 (11.9) | 1016 (23.2) | 1417 (43.6) |
| Arthritis | 4708 (37.4) | 82 (8.4) | 901 (22.7) | 1736 (39.6) | 1989 (61.1) |
| Atrial fibrillation | 12585 (100.0) | 980 (100.0) | 3967 (100.0) | 4385 (100.0) | 3253 (100.0) |
| Cerebrovascular disease | 5047 (40.1) | 78 (8.0) | 928 (23.4) | 1862 (42.5) | 2179 (67.0) |
| Chronic kidney disease | 1380 (11.0) | 17 (1.7) | 195 (4.9) | 442 (10.1) | 726 (22.3) |
| Diabetes | 8662 (68.8) | 270 (27.6) | 2399 (60.5) | 3255 (74.2) | 2738 (84.2) |
| Dizziness | 3387 (26.9) | 11 (1.1) | 336 (8.5) | 1169 (26.7) | 1871 (57.5) |
| Dyspnoea | 313 (2.5) | 1 (0.1) | 36 (0.9) | 100 (2.3) | 176 (5.4) |
| Foot problems | 1120 (8.9) | 11 (1.1) | 168 (4.2) | 400 (9.1) | 541 (16.6) |
| Fragility fracture | 1285 (10.2) | 13 (1.3) | 136 (3.4) | 402 (9.2) | 734 (22.6) |
| Hearing impairment | 1442 (11.5) | 21 (2.1) | 198 (5.0) | 501 (11.4) | 722 (22.2) |
| Heart failure | 8882 (70.6) | 279 (28.5) | 2395 (60.4) | 3355 (76.5) | 2853 (87.7) |
| Heart valve disease | 3661 (29.1) | 80 (8.2) | 862 (21.7) | 1372 (31.3) | 1347 (41.4) |
| Housebound | 181 (1.4) | 0 (0.0) | 15 (0.4) | 39 (0.9) | 127 (3.9) |
| Hypertension | 10365 (82.4) | 582 (59.4) | 3014 (76.0) | 3767 (85.9) | 3002 (92.3) |
| Hypotension/syncope | 3036 (24.1) | 8 (0.8) | 282 (7.1) | 1022 (23.3) | 1724 (53.0) |
| Ischaemic heart disease | 5592 (44.4) | 62 (6.3) | 1112 (28.0) | 2181 (49.7) | 2237 (68.8) |
| Memory and cognitive problems | 2475 (19.7) | 25 (2.6) | 326 (8.2) | 829 (18.9) | 1295 (39.8) |
| Mobility and transfer problems | 1016 (8.1) | 0 (0.0) | 75 (1.9) | 300 (6.8) | 641 (19.7) |
| Osteoporosis | 2924 (23.2) | 26 (2.7) | 391 (9.9) | 1031 (23.5) | 1476 (45.4) |
| Parkinsonism and tremor | 904 (7.2) | 10 (1.0) | 101 (2.5) | 285 (6.5) | 508 (15.6) |
| Peptic ulcer | 8076 (64.2) | 174 (17.8) | 1853 (46.7) | 3149 (71.8) | 2900 (89.1) |
| Peripheral vascular disease | 3479 (27.6) | 23 (2.3) | 524 (13.2) | 1318 (30.1) | 1614 (49.6) |
| Polypharmacy | 7313 (58.1) | 60 (6.1) | 1353 (34.1) | 2967 (67.7) | 2933 (90.2) |
| Requirement for care | 181 (1.4) | 0 (0.0) | 15 (0.4) | 39 (0.9) | 127 (3.9) |
| Respiratory disease | 5860 (46.6) | 135 (13.8) | 1204 (30.4) | 2248 (51.3) | 2273 (69.9) |
| Skin ulcer | 1079 (8.6) | 6 (0.6) | 171 (4.3) | 334 (7.6) | 568 (17.5) |
| Sleep disturbance | 3718 (29.5) | 39 (4.0) | 561 (14.1) | 1383 (31.5) | 1735 (53.3) |
| Social vulnerability | 67 (0.5) | 4 (0.4) | 13 (0.3) | 24 (0.5) | 26 (0.8) |
| Thyroid disease | 4102 (32.6) | 56 (5.7) | 991 (25.0) | 1493 (34.0) | 1562 (48.0) |
| Urinary incontinence | 606 (4.8) | 9 (0.9) | 73 (1.8) | 174 (4.0) | 350 (10.8) |
| Urinary system disease | 2435 (19.3) | 36 (3.7) | 406 (10.2) | 831 (19.0) | 1162 (35.7) |
| Visual impairment | 4004 (31.8) | 104 (10.6) | 835 (21.0) | 1472 (33.6) | 1593 (49.0) |
| Weight loss and anorexia | 1682 (13.4) | 10 (1.0) | 153 (3.9) | 514 (11.7) | 1005 (30.9) |


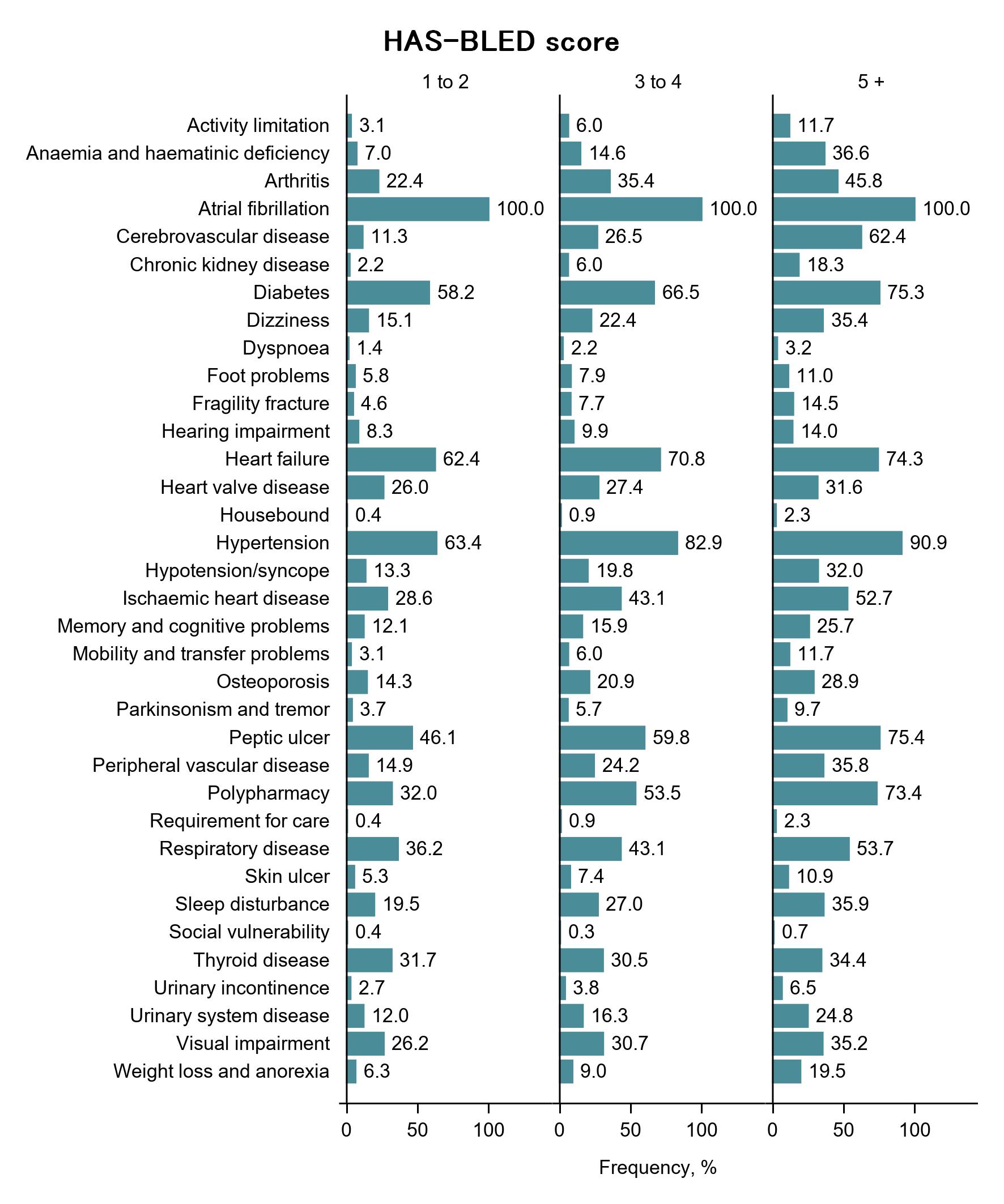


**Supplementary Figure 3.** Prevalence of deficits in eFI by HAS-BLED score group


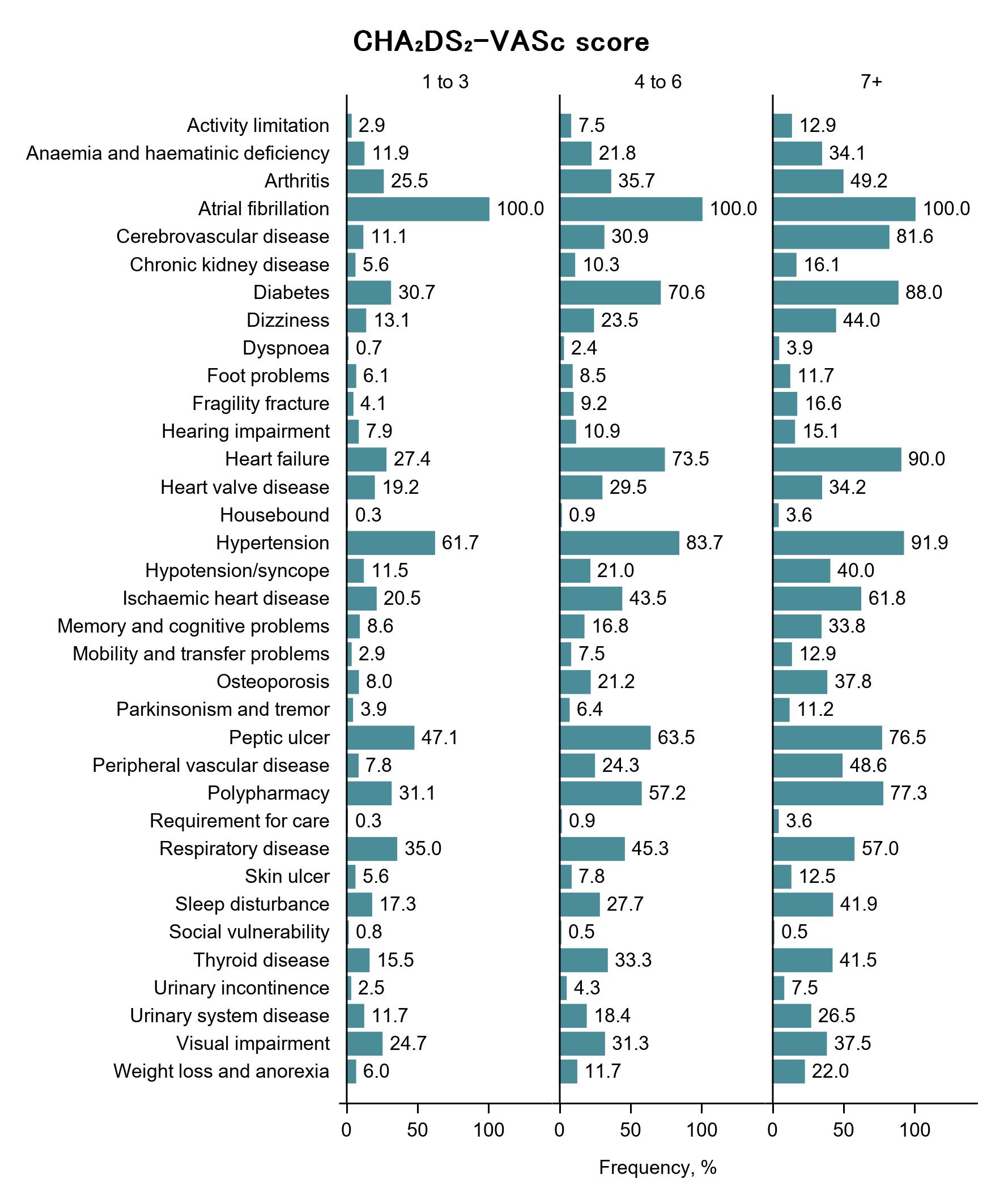


**Supplementary Figure 4.** Prevalence of deficits in eFI by CHA_2_DS_2_-VASc score group


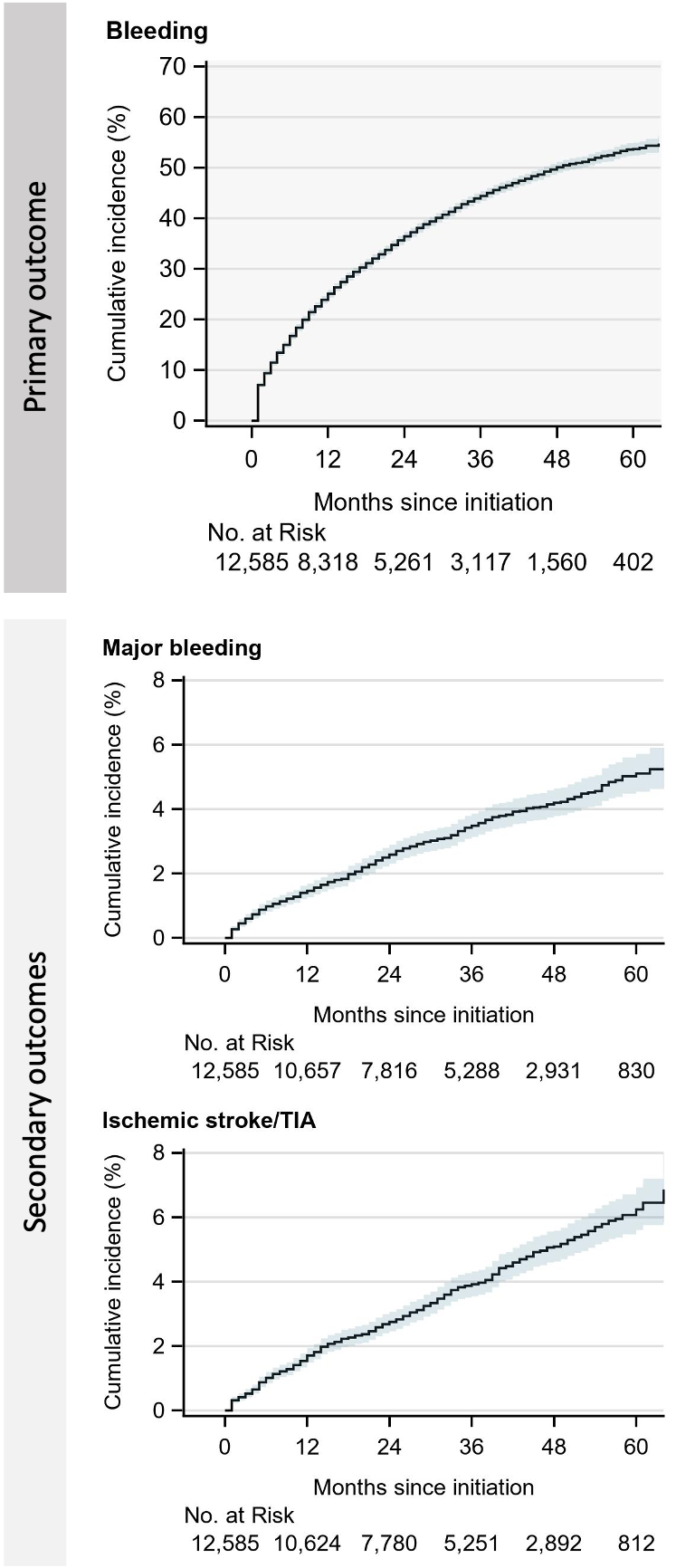


**Supplementary Figure 5.** Cumulative incidence of outcomes by subgroup

Cumulative incidence curves with 95% confidence intervals are plotted.

**Supplementary Table 7.** Association between frailty and outcomes after adjustment for age, sex, baseline comorbidities and medication use

| Outcome | Adjusted^a^ sHR (95% CI) |
| --- | --- |
|  |  |
| **Ischemic stroke/TIA** |  |
| Fit | Ref |
| Mild | 1.16 (0.76–1.77) |
| Moderate | 1.01 (0.64–1.59) |
| Severe | 0.95 (0.58–1.56) |
| **Bleeding** |  |
| Fit | Ref |
| Mild | 1.13 (1.00–1.28) |
| Moderate | 1.36 (1.19–1.55) |
| Severe | 1.75 (1.51–2.03) |
| **Major bleeding** |  |
| Fit | Ref |
| Mild | 1.17 (0.73–1.88) |
| Moderate | 1.27 (0.77–2.10) |
| Severe | 1.18 (0.67–2.07) |
| TIA=transient ischemic attack.  ^a^Models adjusted for age, sex, medical history, and medications. | |

**Supplementary Table 8.** Association between frailty and outcomes after adjustment for sex, baseline comorbidities and medication use with the exception of eFI components that were not related to stroke admission.

| Outcome | Adjusted^b^ sHR (95% CI) |
| --- | --- |
| **Ischemic stroke/TIA** |  |
| Fit | Ref |
| Mild | 1.41 (0.94–2.12.) |
| Moderate | 1.46 (0.97–2.19) |
| Severe | 1.49 (0.97–2.30) |
| **Bleeding** |  |
| Fit | Ref |
| Mild | 1.12 (0.99–1.26) |
| Moderate | 1.36 (1.21–1.53) |
| Severe | 1.79 (1.58–2.03) |
| **Major bleeding** |  |
| Fit | Ref |
| Mild | 1.37 (0.86–2.20) |
| Moderate | 1.76 (1.10–2.83) |
| Severe | 1.83 (1.11–3.02) |
| TIA=transient ischaemic attack.  ^a^Event rate per 100 patient-years.  ^b^Models adjusted for sex, medical history, and medications, with the exception of eFI components that were not related to stroke admission. | |


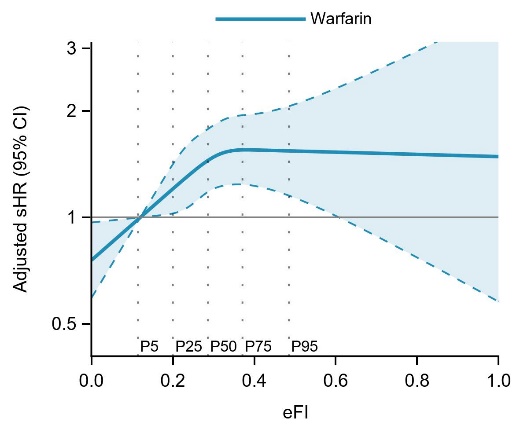

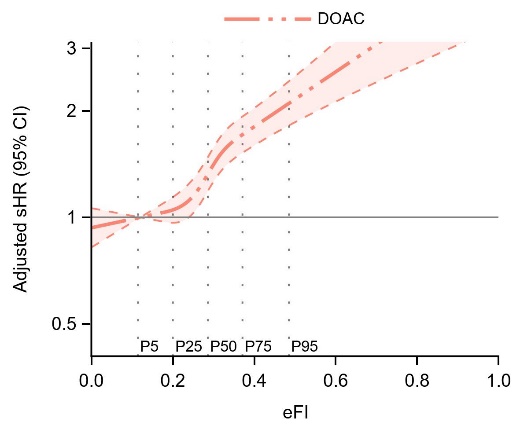


**Supplementary Figure 6.** Associations between eFI and bleeding stratified by warfarin (n=2245) or DOAC (n= 10,340)

*Note:* Model is adjusted for sex, medical history, and medications. Subdistribution hazard ratios with 95% confidence interval are plotted. An eFI score of 0.12 (cut-off score between fit and mild frailty) is the reference standard. Bleeding events are defined as any outpatient or inpatient bleeding event. The vertical dotted lines show thresholds for percentiles of eFI value. Abbreviations: eFI, electronic frailty index; TIA, transient ischaemic attack; DOAC, direct oral anticoagulant; HR, hazard ratio; CI, confidence interval
